# As Hubbard Sees It

**Published:** 1908-06

**Authors:** 


### As Hubbard Sees It.

John Burroughs describes old age as a letting go—a gradual lessening of interest in things which you once thought were vital. And with this letting go should come, and usually does, a glad relief. The fear of poverty, the dread of loss, apprehension concerning social position, the desire for approbation, all these slip away in the person of a well-ordered mind as the years advance. The desire to kill things dies, and all the great living, pulsing world of nature seems a little closer, nearer, dearer, more beautiful. And then for the first time we have leisure to think, contemplate and enjoy. Then for the first time we realize that nothing much matters after all.

Then is the time to enjoy. The individual is cosmopolitan—people interest more than persons.

But this fine life of the intellect can only go with the temperate life, the life of moderation.

Let us look to ourselves for health, not to the doctors. People who are forever taking note of their sensations and who send for a doctor if they feel bad, instead of figuring out in their own minds why they feel bad and avoiding the cause, are candidates for the ether cone. Those who are given to the pleasures of the table are preparing for the pleasures of the operator's table.

The average length of life would be increased immensely if we would just begin to "Know Thyself." As it is now, we depend on the doctors to cure us if we are sick, and if worse comes to worst, we are fully prepared to go to the hospital and have the surgeon remove the inflamed organ. Wouldn't it be better to so live that no inflammation would follow?

Disease comes only to those who have been preparing for it. Disease is a sequence postponed by Nature as long as she can, and then, discouraged, she says "Let 'er go—back to the Mass!"

Beginners on the bicycle run into the object they seek to avoid. The doctor and the hospital are in our minds, we think disease, not happiness and health.

Health is within our reach—it costs nothing—only the effort which soon grows into a pleasurable habit. Ask any doctor of any school if I am not right? Why not acquire the Health Habit?

Here is the formula:

First, deep-breathing in the open air with your mouth closed; second, moderation in eating—simple dishes; third, exercise at least an hour in the open, walking, working in the garden, playing with the children; fourth, sleep eight hours in a thoroughly ventilated room; fifth, drink all the water between meals you care to; sixth, don't bother to forgive your enemies—just forget them; seventh, keep busy—it is a beautiful world, and we must and will and can leave it more beautiful than we found it.—*Elbert Hubbard's The Fra.*

